# Public acceptability of a technology-mediated stool sample collection platform to inform community-based surveillance of infectious intestinal disease: a pilot study

**DOI:** 10.1186/s12889-022-13307-5

**Published:** 2022-05-13

**Authors:** Rowan Davies, Miren Iturriza-Gómara, Rebecca Glennon-Alty, Alex J. Elliot, Roberto Vivancos, Anica Alvarez Nishio, Nigel A. Cunliffe, Daniel Hungerford

**Affiliations:** 1 National Institute for Health and Care Research Health Protection Research Unit in Gastrointestinal Infections, University of Liverpool, Liverpool, UK; 2 School of Medicine, University of Liverpool, Liverpool, UK; 3 Centre for Vaccine Innovation and Access, PATH, Geneva, Switzerland; 4 Department of Clinical Infection, Microbiology and Immunology, Institute of Infection, Veterinary & Ecological Sciences, University of Liverpool, Liverpool, UK; 5 Real-Time Syndromic Surveillance Team, Field Service, Health Protection Operations, UK Health Security Agency, Birmingham, UK; 6 National Institute for Health and Care Research Health Protection Research Unit in Emergency Preparedness and Response, King’s College London, London, UK; 7 Field Epidemiology North West, Field Service, Health Protection Operations, UK Health Security Agency, Liverpool, UK; 8 National Institute for Health and Care Research Health Protection Research Unit in Emerging and Zoonotic Infections, University of Liverpool, Liverpool, UK; 9 Patient and Public Involvement Representative, London, UK

**Keywords:** Gastrointestinal disease, Diarrhoea and vomiting, Surveillance, Application, Smartphone, Patient and public engagement, Survey, Telehealth, Self-reporting, Community

## Abstract

**Background:**

In the UK approximately a quarter of the population experience infectious intestinal disease (IID) each year. However, only 2% present to primary care, preventing a true determination of community burden and pathogen aetiology. The aim of this pilot study was to gauge public acceptability of a technology-mediated platform for reporting episodes of IID and for providing stool samples.

**Methods:**

This study employed a cross-sectional online survey design, targeting individuals 16 + years old within Liverpool City Region, UK. Information sought included demographics, comfortability of reporting illness and IID symptoms, willingness to provide stool, and favoured stool-provision method. Univariable logistic regression was used to examine associations between demographic variables and providing a stool sample. Odds ratios (OR) and associated 95% confidence intervals (CIs) were produced.

**Results:**

A total of 174 eligible participants completed the survey, with 69% female. The sample was skewed towards younger populations, with 2.9% aged 65 + years. Nearly a third (29%) had a household income of less than £30,000 per annum and 70% had attained a degree or higher. The majority identified as White British (81%) and 11% identified as ethnicities typically grouped Black, Asian and minority ethnic (BAME). Three quarters of participants were either ‘Comfortable’ or ‘Very Comfortable’ with reporting illness (75%) and with answering symptom-related questions (79%); 78% reported that they would provide a stool sample. Upon univariable analysis, increasing age – being 55 + (OR 6.28, 95% CI 1.15–117.48), and lower income (OR 2.5, 95% CI 1.02–6.60), was associated with willingness to provide a stool sample. Additionally, respondents identifying as BAME ethnicities and men may be less inclined to provide a stool sample.

**Conclusions:**

This pilot study assessed the acceptability of technology-mediated platforms for reporting IID and provision of stool samples in the community. Respondents were biased towards younger, technologically inclined, more affluent and educated populations. Acceptability for reporting illness and providing a stool sample through technology-mediated platforms was high. While older populations were under-represented, they were more likely to agree to provide a stool sample. Qualitative research is required to better reach older and more deprived populations, and to understand potential age, gender and ethnic differences in compliance with stool sampling.

**Supplementary Information:**

The online version contains supplementary material available at 10.1186/s12889-022-13307-5.

## Background

Infectious intestinal disease (IID) is a major cause of morbidity among populations worldwide. According to the second Infectious Intestinal Disease (IID2) study [1], approximately 25% of the UK population suffer an episode of IID (diarrhoea and/or vomiting) each year – equivalent to 17 million cases annually. The IID2 study, the most recent comprehensive analysis of IID in the UK community, also identified that around 50% of people with IID reported absence from school or work, equating to nearly 11 million days lost among people of working age. Previous studies in the UK identified norovirus, rotavirus, sapovirus*,* and *Campylobacter* [1, 2] as the most common pathogens responsible for community IID; although, rotavirus prevalence has dramatically reduced since the introduction of rotavirus vaccination in 2013 [3].

Despite the ubiquitous community presence of IID, our understanding of the microorganisms responsible for these symptoms is comparatively limited. A quarter of the population suffer with IID symptoms each year, but only 2% of the cases present to primary care [1], and fewer still are reported to national surveillance programmes. Therefore, faecal samples are obtained from a minority of those with disease and are typically not representative of communities or populations. This limited sampling in turn limits our ability to monitor seasonal activity, timely detection of new strains and deprives us of early warning in institutional outbreaks.

A self-conducted symptom reporting and stool sampling system within the community may improve the recording of cases and facilitate faecal sampling. However, public participation in such programmes is reported to be poor [4, 6]. Lecky et al. highlighted problems of recruitment to a community-based survey of resistant bacterial carriage in England; complete return of both stool sample and questionnaire varied by area, age, gender and ethnicity, with the overall return rate being only 3.9% [4]. The authors considered that a pertinent factor to explain variable participation was a lack of perceived health benefit to the individual. Ellis et al. examined factors influencing self-collection of stool samples for bowel cancer screening [5]. Perceived unpleasantness of collecting and potentially storing of one’s own faecal material significantly affected compliance [7, 8]. Studies have suggested that uptake may be enhanced by emphasising the convenience of testing kits and providing instructions on easy collection/storage methods at the start of the process [9].

There is a lack of information on how the usage of technology-based platforms for community IID surveillance and self-conducted stool sampling could influence public engagement with such programmes. We aimed to develop and deliver a questionnaire that assesses the acceptability of a mobile application/secure website/text message platform for members of the public to report an episode of IID and request a stool sample collection kit for use and return to a laboratory.

## Methodology

### Population and setting

The target population comprised adults (16 + years of age) residing in the Liverpool City Region. Liverpool City Region contains six local authority areas (Halton, Knowsley, Liverpool, St. Helens, Sefton and Wirral) and has an estimated resident population of approximately 1.43 million [10] [Table 1]. The region includes some of the most socioeconomically deprived communities in England, and some neighbourhoods which are among the most affluent [11].

**Table 1:** Basic demographic details for local authorities in Liverpool City Region, and England (derived from the Office of National Statistics) [11–15]

Local Authority	% White British	Mean Household Income (£)	% Age > 65
Halton	96.8	37,475	18.4
Knowsley	95.3	33,205	17.3
Liverpool	82.8	33,724	14.8
St. Helens	96.6	37,726	20.6
Sefton	94.9	38,363	23.6
Wirral	94.4	39,207	21.9
*England*	*78.4*	*34,200*	*18.5*

### Study design and questionnaire

The study utilised a cross-sectional community survey conducted between December 2020 and March 2021. All data were collected via an online questionnaire designed and delivered using ‘JISC Online Surveys’ (Additional File 1) [16]. A summary of the study’s purpose, inclusion criteria (adults 16 + years of age resident in Liverpool City Region), voluntary nature, confidentiality and right to withdraw was presented prior to obtaining participant informed valid consent to complete the survey.

Participants were asked to provide anonymous information to establish demographic characteristics including age, gender, ethnicity, highest level of education, postcode district and household income. Options for ethnicity were given in accordance with the Office for National Statistics (ONS) classification system for coding ethnicity in the UK [12]. The following sections of the questionnaire then asked questions pertaining to the acceptability of the various stages of engaging with this service. The remainder of the questionnaire was split into four sections: ‘Provision of data (concerning illness)’, ‘Poo (stool) sample collection’, ‘Poo (stool) sample storage and return’ and ‘Receiving results’.

The question response format primarily comprised a 5-point ordinal scale (Likert scale), consistent with previous questionnaires aiming to ascertain public opinion regarding community stool sampling [5, 10]. Responses were in most cases a choice of a 1–5 graded answer, with 1 representing ‘Very comfortable/Strongly agree/Very likely’ and 5 representing ‘Very uncomfortable/Strongly disagree/Very unlikely’. Two questions had binary outcomes; whether they would provide stool (yes/no), and their preferred method of stool sampling (Method A or Method B). Method A presented the instructions for the current FIT kit used for bowel cancer screening in the National Health Service (NHS) [17] and involved using a container or layers of toilet paper to catch stool first before sampling with a small scoop attached to the inside lid of the sample tube. Method B presented the use of a faecal collection device [https://www.faecal-immunochemical-test.co.uk/products/faecal-collection/], which utilises a biodegradable ‘poo hammock’ device which can be attached to the toilet seat to catch stool before sampling in the same way as Method A. The remaining questions were aimed at determining participant motives for agreeing or disagreeing with a previous statement/question. These few questions featured dropdown choices of common causal factors i.e. when disagreeing with providing a stool sample, options included ‘perceived unpleasantness of the activity’ or ‘too time-consuming’.

Both the structure and the terminology used in the questionnaire was refined following review by a patient and public involvement (PPI) panel, so as to encourage maximum response from participants. Following completion of the questionnaire, participants were presented with a link to the NHS information on diarrhoea and vomiting [18].

### Recruitment

Participants were recruited through multiple online sources to capture the most demographically diverse range of participants possible. These sources included: adverts to community centres via email and social media (Facebook message); sharing by local council groups on webpages and social media; distribution through National Institute for Health and Care Research (NIHR) Collaboration for Leadership in Applied Health Research and Care (CLAHRC, now ARC) contributors; University of Liverpool email/webpages for staff and students; and social media followers of the Faculty of Health and Life Sciences.

### Data analysis

All analyses were conducted using R and Rstudio (Version 1.4.1106). We excluded from the analysis those participants who provided consent only for demographic variables, and those who entered a first part postcode from outside of Liverpool City Region.

Due to the small sample size, all those persons identifying as non-white British white ethnicities, including white other, Irish, Gypsy or Irish Traveller were coded into the larger group ‘White Other’. Persons identifying as non-white ethnicities, including: ‘Mixed/Multiple Ethnic Groups’; White and Asian, White and Black African, White and Black Caribbean, Other; ‘Asian British/Asian’; Chinese, Pakistani, Indian, Bangladeshi, Other; ‘Black British/Black’, African, Caribbean, Other; and ‘Other Ethnic Group’; Arab, Other were coded into the larger group ‘Black, Asian and minority ethnic’ (BAME). Similarly, amongst ‘Number of Children’, all responses with a number of children of 3 or higher were coded into the larger group ‘3 or more’ and all responses with a number of children that was 1 or 2 were coded into the larger group ‘1–2’.

Descriptive analysis of the responses including the variables of interest was conducted and summary statistics calculated and tabulated.

Univariable logistic regression analysis was conducted to assess any associations between demographics/background questions (independent variables) and whether a participant would be happy to provide a stool sample (dependent: yes/no). Some categories were collapsed for the purposes of the univariable analysis. Age groups ‘55–64’ and ‘65 + ’ were aggregated to ‘55 + ’ and ‘16–17’ was included with ‘18–24’ to form the group ‘16–24’. Household Income groups ‘ < £10,000’, ‘£10,001–20,000’ and ‘£20,001–30,000’ were collapsed to ‘ < £30,000’, as well as ‘30,001–45,000’ and ‘£45,001–60,000’ to ‘£30,000–60,000’. Educational Attainment groups ‘Masters’ and ‘PhD’ were collapsed to ‘Masters/PhD’, ‘NVQ/GCSE/O Level’ and ‘A levels/BTEC/level 3 diploma’ were collapsed to ‘Lower than Degree’, as well as ‘Degree/PGDips’ and ‘Other’ to ‘Degree/HND/PGDips’. The models enabled the calculation of odds ratios (ORs) and robust 95% confidence intervals (CIs).

### Ethics, consent and data privacy

The survey was anonymous, as participants did not have to provide names, date of birth or contact information. Valid informed consent was gained from study participants prior to completion of the questionnaire; recorded using clear affirmative action by use of a consent checkbox. Participants who did not tick the consent box were not included in the subsequent data analysis and only adults aged 16 + years were eligible. All methods were performed in accordance with the relevant guidelines and regulations and conformed to the general ethical principles of Helsinki Declaration. The study received ethical approval from the University of Liverpool Health and Life Sciences Research Ethics Committee (Human participants, tissues and databases) (approval number: HLS-7962).

## Results

In total, 182 individuals responded to the survey, of whom, two did not provide consent for their data to be analysed and a further six participants had first part postcodes which indicated they were not living in the designated study area. These eight individuals were excluded from the analysis, providing a final sample of 174 participants.

### Sample characteristics

**Fig. 1:**
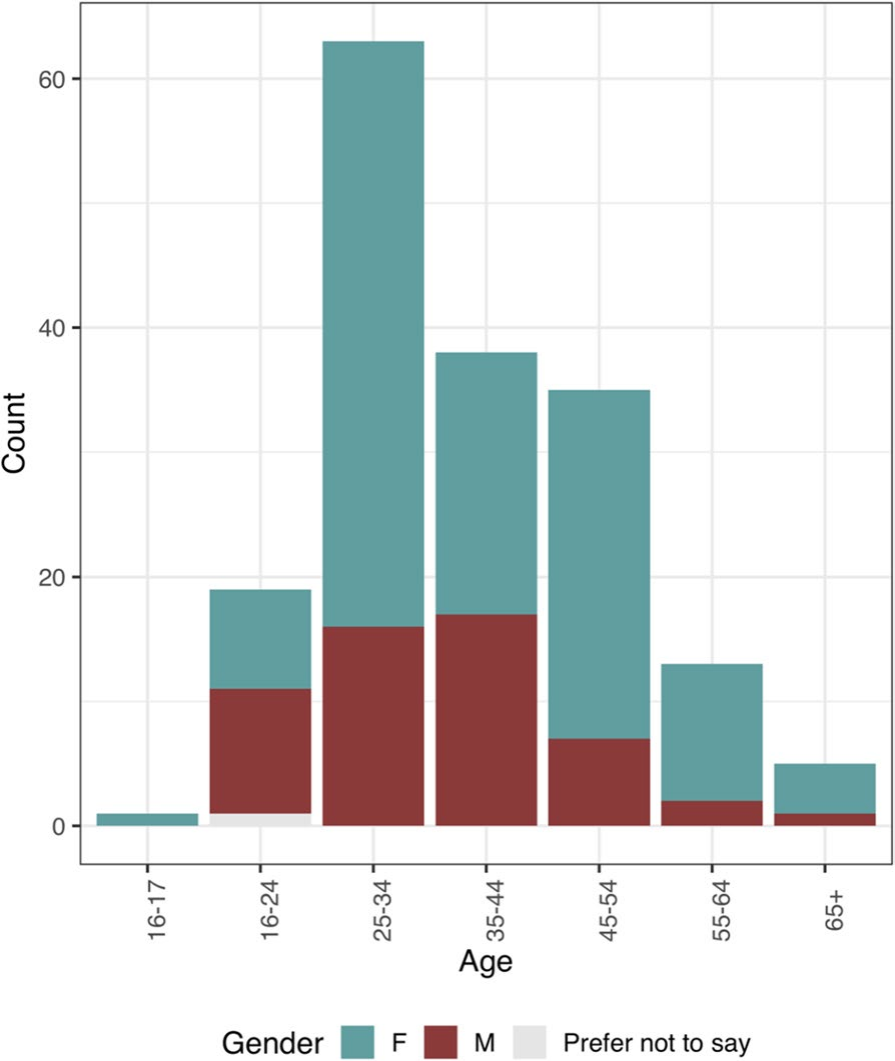
Number of respondents in each age group, and gender

The majority of participants were female (69%) (Table 2, Fig. 1). The majority of the sample was represented by younger age groups, in particular 25–34 year olds (36.2% of the total), with 89.6% being < 55 years, and only 2.9% being > 65 years. Ethnicity in the sample was approximately representative of the wider Liverpool region (Table 1), with 89% identifying as White (including British, Irish, Gypsy or Irish Traveller, Other). More socioeconomically advantaged groups were over-represented, with 70.7% of the sample having attained a Degree or higher and 32.8% having a household income of greater than £60,000 per annum. The entire sample owned a smartphone. Over a third (37.9%) of the sample did not access their General Practitioner (GP) online for any service. In those that did access their GP online, the most common reasons for accessing their GP online were, booking appointments (76.9%) and requesting prescriptions (65.7%).

**Table 2:** Survey Participant demographics

Demographic Variables	Overall (*N* = 174)
**Gender**	**(*N* = 174)**
Female	120 (69.0%)
Male	53 (30.5%)
Prefer not to say	1 (0.6%)
**Age group (in years)**	**(*N* = 174)**
16–17	1 (0.6%)
18–24	19 (10.9%)
25–34	63 (36.2%)
35–44	38 (21.8%)
45–54	35 (20.1%)
55–64	13 (7.5%)
65 +	5 (2.9%)
**Household Income**	**(*N* = 172)**
< £10,000	9 (5.2%)
£10,001–20,000	10 (5.7%)
£20,001–30,000	32 (18.4%)
£30,001–45,000	29 (16.7%)
£45,001–60,000	35 (20.1%)
> £60,000	57 (32.8%)
**Educational Attainment**	**(*N* = 173)**
A levels/BTEC/level 3 diploma	36 (20.7%)
Degree/PGDips	68 (39.1%)
Masters	35 (20.1%)
NVQ/GCSE/O Level	12 (6.9%)
Other	2 (1.1%)
PhD	20 (11.5%)
**Ethnicity**	**(*N* = 174)**
BAME^b^	19 (10.9%)
White British	141 (81.0%)
White Other^a^	14 (8.0%)
**Number of Children**	**(*N* = 168)**
0	102 (58.6%)
1 to 2	59 (33.9%)
3 or more	7 (4.0%)

Proportions expressed as percentages account for (include) missing data from survey

^a^White Other: included those who identified as; Irish, Gypsy or Irish Traveller, Other,

^b^BAME: included those who identified as; ‘Mixed/Multiple Ethnic Groups’; White and Asian, White and Black African, White and Black Caribbean, Other; ‘Asian British/Asian’; Chinese, Pakistani, Indian, Bangladeshi, Other; ‘Black British/Black’; African, Caribbean, Other; ‘Other Ethnic Group’; Arab, Other

The majority of the sample responded as being either ‘comfortable’ or ‘very comfortable’ (74.8%) with reporting an episode of illness to a non-GP based, technology-mediated service (Table 3). A similar proportion was either ‘comfortable’ or ‘very comfortable’ with answering additional questions related to their symptoms (78.8%).

**Table 3:** Participant responses to each of the survey’s main outcomes

Main Survey Outcomes	Overall *N* = 174
**Comfortability Reporting Illness Online**	***N* = 173**
Very Comfortable	61 (35.1%)
Comfortable	69 (39.7%)
Neither Comfortable or Uncomfortable	22 (12.6%)
Uncomfortable	20 (11.5%)
Very Uncomfortable	1 (0.6%)
**Comfortability Describing Symptoms Online**	***N* = 172**
Very Comfortable	64 (36.8%)
Comfortable	73 (42.0%)
Neither Comfortable or Uncomfortable	21 (12.1%)
Uncomfortable	14 (8.0%)
**Would provide a stool sample if asked**	***N* = 174**
No	39 (22.4%)
Yes	135 (77.6%)
**If No to stool; what factor might change your mind? (multi-response)**	***N* = 39**
Being provided with ‘diagnosis’ of causative microbes	16 (41%)
Helping to aid in scientific research	6 (15.4%)
Helping to identify disease outbreaks	11 (28.2%)
Other	1 (2.6%)
**Chosen Stool Collection Method**^a^	***N* = 169**
Method A	115 (66.1%)
Method B	54 (31.0%)
**Wanted Informing of Test Outcome**	***N* = 173**
No	3 (1.7%)
Yes	170 (97.7%)
**Wanted further information on microbes**	***N* = 173**
No	5 (2.9%)
Yes	168 (96.6%)

Proportions expressed as percentages account for (include) missing data from survey

^a^Method A—presented the instructions for the current FIT kit used for bowel cancer screening in the National Health Service (NHS) [17], and Method B—presented the use of the Fe-Col® faecal collection device

Over three quarters (77.6%) of the sample said they would provide a stool sample if prompted (Table 3). Method A was the preferred method of stool sample collection (66.1%). This was the method that involved direct sampling of stool, rather than utilising a ‘poo hammock’ as in Method B (Appendix 1). Among those who stated that they would refuse to provide a stool sample, more than 40% said that they could change their mind, most commonly through being provided with a ‘diagnosis’ of causative microbes.

Almost all (97.7%) of respondents indicated that they would want to be informed of the results of the tests carried out on their stool sample. Similarly, almost all (96.6%) respondents said they would want more information about the microbe if one was identified. Generally, the number of respondents (N) to questions throughout the survey was relatively consistent.

In descriptive analysis of stool sample provision, men were less likely to provide a stool sample than women (Table 4). When compared with those who identified as White British, those who identified with BAME ethnicities were less likely to provide a stool sample and those who identified as other White ethnicities were more likely to provide a stool sample. Additionally, those with the highest educational attainment were more likely to provide a stool sample.

**Table 4:** Univariable analysis of the association between demographic factors and providing a stool sample

Independent variable	Would provide a stool sample		Univariable Analysis		
	**No (%)**	**Yes (%)**	**OR**	**95% CI**	**P value**
Age group (25–34 years)	17 (43.6%)	46 (34.1%)	Ref	NA	NA
Age group (16–24 years)	7 (17.9%)	13 (9.6%)	0.8	0.27–2.58	0.696
Age group (35–44 years)	9 (23.1%)	29 (21.5%)	1.19	0.48–3.12	0.713
Age group (45–54 years)	5 (12.8%)	30 (22.2%)	2.22	0.78–7.32	0.155
Age group (55 + years)	1 (2.6%)	17 (12.6%)	6.28	1.15–117.48	0.085
Gender (Female)	24 (61.5%)	96 (71.1%)	Ref	NA	NA
Gender (Male)	14 (35.9%)	39 (28.9%)	0.7	0.33–1.51	0.349
Income (£30,001–60,000)	21 (53.8%)	43 (31.9%)	Ref		
Income (< £30,000)	8 (20.5%)	43 (31.9%)	2.50	1.02–6.6	0.052
Income (> £60,000)	10 (25.6%)	47 (34.8%)	2.28	0.98–5.58	0.061
Education (Degree/HND PGDips)	17 (43.6%)	53 (39.3%)	REF	NA	NA
Education (< Degree)	14 (35.9%)	34 (25.2%)	0.84	0.36–1.97	0.682
Education (Masters/PhD)	8 (20.5%)	47 (34.8%)	1.88	0.76–4.99	0.181
Ethnicity (White British)	31 (79.5%)	110 (81.5%)	REF	NA	NA
Ethnicity (BAME)	6 (15.4%)	13 (9.6%)	0.61	0.22–1.86	0.355
Ethnicity (White Other)	2 (5.1%)	12 (8.9%)	3.38	0.63–62.8	0.251
No. of Children (0)	21 (53.8%)	81 (60.0%)	REF	NA	NA
No. of Children (1–2)	15 (38.5%)	44 (32.6%)	0.72	0.34–1.57	0.723
No. of Children (3 +)	1 (2.6%)	6 (4.4%)	1.48	0.23–28.81	0.408

*OR* odds ratio, *CI* confidence interval

Upon univariable logistic regression, only age and income were associated with agreeing to provide a stool sample if asked (Table 4). The variable with the strongest association was age, with those more than 55 years old having more than 6 times the odds of providing a stool sample compared to those aged 25–34 (OR 6.28, 95% CI 1.15–117.48; p = 0.085). Those with a household income less than £30,000 (OR 2.5, 95% CI 1.02–6.6; p = 0.052) and those with a household income of more than £60,000 (OR 2.28, 95% CI 0.98–5.58; p = 0.061), had more than twice the odds of providing a stool sample than those whose household income was £30,000-£60,000.

## Discussion

This is one of the first studies to address attitudes towards technology-mediated reporting of illness and provision of stool samples in the UK. The majority of respondents were either comfortable or very comfortable with reporting illness to a non-GP based online platform, and most said they would provide a stool sample if they were asked for one. This is one of the first reports of acceptability of stool sampling which is not directly related to a perceived personal health benefit (such as Faecal Occult Blood Testing – reported 94.5% acceptability) [5].

While those who said they would refuse to provide a sample indicated that their main reason for doing so would be due to the ‘unpleasantness of stool collection’, which is a more common reason than previously reported [5, 7, 8], it was interesting to note the overall high degree of acceptability. There may be several explanations for these findings. Firstly, our sample was small and skewed towards more affluent and educated participants, who are generally considered to be more engaged with research. This is likely because the survey was advertised on university networks. Secondly, opinion may have been influenced due to our survey being open during the COVID-19 pandemic. This may be due to the population’s new familiarity with general testing procedures and receipt of results, as well as awareness of microbes and infectious disease. There is further support for this in our findings regarding participants’ motivations to provide a sample or factors which may change their mind, with the commonest answer being ‘being provided with a diagnosis of causative microbes’, and almost all indicating they would like more information about the organisms detected in their stool. Regardless of whether this attitude was influenced by COVID-19, this strongly suggests that the inclusion of a ‘test result and associated information’ feature would be beneficial in any future online service. It is also evident that we have accessed a population that is technologically inclined, with all participants owning a smartphone and the majority accessing their GP online for at least one service. This is likely to make this sample more trusting of technological platforms and not generalisable to the wider population.

The sample was not entirely representative of the population of the Liverpool City Region. The most underrepresented groups those of more deprived background (based on educational attainment and income), and those older than 65. There were fewer responses from those identifying as males compared to females. Since this survey was circulated via online means only, due to the restrictions of the COVID-19 pandemic, older populations were likely not as well accessed. It has been repeatedly reported that acceptance of online technology [19] and engagement with online health services, such as accessing GPs, declines sharply in the oldest age groups [20, 21]. The method of distribution also likely explains why all participants owned a smartphone. However, it could be argued that this reflects the benefits of the proposed online service. We know that reporting episodes of IID to the GP is rare [1], but that older age groups are more likely to report directly to their GP when they experience bouts of IID. This suggests that an online service could ‘catch’ the infections we usually miss, particularly those that are self-limiting (e.g. resolved without treatment) in the younger population. This could indicate an opportunity to identify a proportion of IID which normally goes unreported. However, socioeconomic factors may influence the acceptability of the stool sampling process indicated in the younger age groups sampled; deprivation gradients have been observed with engagement with online health services, such as appointment booking in general practice [20].

It is widely accepted that those who are less socioeconomically deprived and those with higher educational attainment tend to benefit from better health and health awareness than the majority of the population. This should influence future consideration of introducing a technology-mediated IID reporting and stool sampling service, as the overall benefit of developing such a service may be reduced if it only succeeds in engaging with these groups who are already adept at accessing health services.

### Strengths and Limitations of the study

Given that the survey aimed to represent the Liverpool City Region, the demographics of the sample strongly suggest that distribution methods require addressing in future work. One factor was the absence of active or in-person collection methods, which is likely to have returned more representative data. However, more careful targeting is required for optimal results [22]. Our institute PPI panel has suggested this could be achieved by organising focus groups, recruiting from local amateur sports clubs, breakfast clubs, veteran’s organisations, faith groups, food banks, housing offices and public houses, for example. They could contribute to the design of the questionnaire, specify preferred methods of engagement, or suggest more appropriate distribution methods.

A greater sample size would have been desirable to allow for more advanced statistical analysis of the survey results. Multivariable analysis of demographic variables in relation to providing a stool sample was intended. However, this assumed a 50/50 split for the stool sampling response. This may have enabled identification of adjusted, significant contributing factors to the main outcome variable. Advertising and active collection of survey responses in public spaces had also initially been intended, however, the study coincided with national and regional COVID-19 population lockdowns, which hindered the survey being shared in this fashion. This could be remedied in future studies by keeping the survey open for longer and distributing the survey through more varied and targeted means, as aforementioned.

When considering engagement from the participants, the N value remained fairly consistent throughout the survey (where all participants were expected to respond). This suggests that the survey was designed in a way that encouraged full and complete responses, chiefly in its length. Finally, although the method of distribution hindered the study in some respects, it was likely the most situationally appropriate method that could have been used at the time, given the ongoing pandemic-related restrictions.

## Conclusions

In a small sample that was under-representative of some key demographic groups, the study found a high acceptability (78%) of a technology-mediated community-based IID surveillance with patient-led stool sample collection service. This may be due to the skew of the sample towards being younger, more affluent and educated participants. Paradoxically, this pilot study therefore supports the utility of such as a service, as it has already been identified that an increasing number of young people do not report IID to their GP. This indicates that a service of this nature may help improve surveillance systems by capturing a large proportion of IID currently going unreported in the community. The study also highlights what is being discovered across much technology-mediated healthcare, which is that one default system does not suit all situations [23, 24]. It therefore suggests the approach should be used in conjunction with existing GP-based surveillance, as well as other approaches that target groups less likely to use technology to access health services. These results support a definitive study using a larger, more representative sample, informed by qualitative targeted research in the community. In this way, future research might elucidate whether this degree of acceptability would be observed in the more deprived groups. It would also be advisable to investigate how proposed changes would affect GP workload. Finally, this study also highlights and strengthens the need for introducing future IID community-based surveillance schemes, potentially underpinned by self-sampling, which would improve our understanding of the true (and changing) burden of IID pathogens in the community.

## Supplementary Information

**Additional file 1.** Questionnaire: Acceptability of a technology-mediated way to record diarrhoea illness.

## Data Availability

The data in this study was collected via a survey from members of the public. As the study participants did not explicitly consent to their data being made available in a public repository, we have been advised by the University of Liverpool Research Data Management Team (rdm@liverpool.ac.uk) that it is not appropriate to make the data available in this format. However, datasets used and/or analysed during the current study may be available from the corresponding author on reasonable request.

## Abbreviations

BAME: Black, Asian and minority ethnic
CI: Confidence Interval
GP: General Practitioner
IID: Infectious Intestinal Disease
NHS: National Health Service
OR: Odds Ratio
ONS: Office for National Statistics
PPI: Patient and Public Involvement
UK: United Kingdom
