# Supplementary material for: Public acceptability of a technology-mediated stool sample collection platform to inform community-based surveillance of infectious intestinal disease: a pilot study
Source: BMC Public Health. 2022 May 13;22:958. doi: 10.1186/s12889-022-13307-5 (PMC9099322; doi:10.1186/s12889-022-13307-5)
Supplement: Supplementary file 1 — Additional file 1. Questionnaire: Acceptability of a technology-mediated way to record diarrhoea illness. [file 12889_2022_13307_MOESM1_ESM.pdf]

Acceptability of a technology-mediated way to record diarrhoea illness

Page 1: Introduction

This is part of a research project to explore new ways of finding out which bugs (and how often these bugs) cause diarrhoea disease in the community.

The purpose of this survey is to understand how members of the public, such as yourself, might feel about reporting episodes of diarrhoea through a technology-based service (e.g. via text message, a website or mobile-app).

This WOULD NOT replace your GP service or NHS 111 telephone services that you would normally use if you were concerned that you are unwell.

We believe it would instead offer:

- an opportunity to understand how often and how severely people are affected by diarrhoea.
- an opportunity for Public Health England, the NHS and University teams to collect information on gut health and infections in the community;
- individuals the chance to find out which bug may be causing their diarrhoea

This survey should only take **less than 10 minutes** to complete and once finished you will be asked if you want to enter a **prize draw** for a chance to win a **£100 amazon voucher.**

All of the responses to this questionnaire are confidential and anonymous (no information will be gathered that could identify you). Participation in this questionnaire is completely voluntary and you may choose to leave the survey at any time if you feel you do not wish to complete it. Since the questionnaire is confidential, once submitted, no responses can be withdrawn as it will not be possible to find which responses relate to you. Data collected from this questionnaire will be retained for 10 years on the secure University network and then deleted.

This questionnaire is voluntary and as such, if a question makes you uncomfortable, you may choose to leave it blank. However, we ask that you answer as many of the questions as you can.

Please only complete this questionnaire if you live in the Liverpool City Region (Liverpool, Knowsley, Sefton, St Helens, Wirral and Halton).

If you have already fully completed this questionnaire, please exit the survey.

If you have further questions please contact:

Dr Dan Hungerford

NIHR Health Protection Research Unit in Gastrointestinal Infections

University of Liverpool,

Email: d.hungerford@liverpool.ac.uk | Telephone: 0151 795 1455

Please tick/click the box below to indicate your consent to the above:

- [ ] I consent to take part in the above study

Page 2: Demographics

Questionnaire: Acceptability of a technology-mediated (e.g. via text message, a website or mobile-app) way to record diarrhoea.

Thank you for agreeing to fill out this questionnaire. Your thoughts and opinions will be helpful in order to guide future research.

What gender do you identify as?

- Male (including transgender men)
- Female (including transgender women)
- Prefer not to say
- Prefer to self-describe

If you selected Other, please specify:

What is your age in years?

What is your highest educational attainment?

If you selected Other, please specify:

What is your current occupation?

How many children do you have at home with you?

Please enter a whole number (integer).

What is your estimated family income (before tax)?

- <£10,000
- £10,001-20,000
- £20,001-30,000
- £30,001-45,000
- £45,001-60,000
- >£60,000

What is your ethnicity?

Do you own a smartphone?

- Yes
- No

What is the first part of your postcode? (e.g. L17)

Do you access your GP online for any of these services? (check all that apply)

- Book appointments
- Request prescriptions
- Access medical records

☐ Do not access my GP online ☐ Other

If you selected Other, please specify:

Page 3: Provision of Data

For this section assume you have experienced a bout of diarrhoea, gastroenteritis or change in bowel habit.

To what degree would you feel comfortable reporting an episode of illness (diarrhoea) to a non-GP based, technology-mediated service (e.g. via text message, a website or mobile-app)?

Please don't select more than 1 answer(s) per row.

	Very comfortable	Comfortable	Neither Comfortable or Uncomfortable	Uncomfortable	Very Uncomfortable
Select one	☐	☐	☐	☐	☐

If you were going to use a technology-mediated service to report diarrhoea illness, which format would you prefer?

- ○ Text message-based
- ○ Secure website-based
- ○ Mobile app-based

To what degree would you feel comfortable answering questions relating to your symptoms of diarrhoea (e.g, duration of illness or description of poo)?

Please don't select more than 1 answer(s) per row.

	Very comfortable	Comfortable	Neither Comfortable or Uncomfortable	Uncomfortable	Very Uncomfortable
Select one	☐	☐	☐	☐	☐

To what degree would you feel comfortable answering questions relating to places (e.g. restaurants or travel abroad) or people (e.g. ill colleagues or family members) you may have been in contact with around the time you were ill but without naming them?

Please don't select more than 1 answer(s) per row.

	Very comfortable	Comfortable	Neither Comfortable or Uncomfortable	Uncomfortable	Very Uncomfortable
Select one	☐	☐	☐	☐	☐

If you felt uncomfortable with the thought of answering these questions, please indicate why below.

If you were to use a technology-mediated service, where would you expect to find out about it? (e.g. GP surgery, via social media, posters in hospital etc.)

Page 4: Poo (stool) sample Collection

For this section, please assume you have contacted the service and provided the information you are comfortable with providing.

If we asked for a poo (stool) sample, would you provide one?

- Yes
- No

If you answered 'No' to the previous question, what would be your main reason?

- Unpleasantness of stool collection
- Concerns over storing your sample
- Concerns over sending your stool sample
- Too time consuming
- Other

If you selected Other, please specify:

If 'No' Would any of the following motivating factors change your mind?

- Being provided with 'diagnosis' of causative microbes
- Helping to aid in scientific research
- Helping to identify disease outbreaks
- Other

If you selected Other, please specify:

If 'Yes', what would be your main motivating factor?

- Being provided with 'diagnosis' of causative microbes

☐ Helping to aid in scientific research

☐ Other

If you selected Other, please specify:

Please view the below descriptions of methods of stool collection, ***please assume you were willing to provide a stool sample***. Which is your preferred method of sample collection?

○ Method A

○ Method B

Method A

https://www.gov.uk/government/publications/bowel-cancer-screening-kit-how-to-use/nhs-bowel-cancer-screening-fit-kit-instructions

Method B

https://www.faecal-immunochemical-test.co.uk/products/faecal-collection/

Biodegradable paper provided in the kit which can be securely looped over the toilet seat to catch the stool.

Stool (poo) can then be collect using a spatula or stick and placed in the sample tube like the method above. When finished with the biodegradble paper can be torn at the sides and flushed down the toilet bowl.

Page 5: Storage and Return

For this section, please assume you have agreed to provide a stool sample.

If you were unable to post the sample straight away would you feel comortable storing it in any of the following:

- ☐ A cool, dark place indoors
- ☐ A cool, dark place outdoors
- ☐ The fridge in a sealed plastic bag
- ☐ None

Would you feel comfortable posting this sample to a testing lab using a pre-addressed envelope?

- ○ Yes
- ○ No

Page 6: Receiving Results

For this section, please assume that you have collected and returned a stool sample by your preferred methods.

Would you like to know the outcome of the tests carried out on your stool sample?

- Yes
- No

If 'Yes', how would like to be informed?

- Via the technological service (e.g text, or by app notification), no matter the result
- Via the technological service (e.g text, or by app notification), only if there was a conclusive result.

If a bug (e.g. virus or bacteria) was identified in your stool sample, would you like to receive additional information about the bug identified?

- Yes
- No

If 'Yes', how would you like to receive this information?

- Receive a website address link to an information page on the microbe
- Receive a short paragraph giving some brief information on the microbe (within the text/website/mobile app service)
- Other

If you selected Other, please specify:

If a bug (e.g. virus or bacteria) was identified in your stool sample would you wish to consult your GP?

- Yes
- No

Page 7

How did you receive this survey? (Tick all that apply)

- [ ] Email from your instituition
- [ ] Social media (e.g. Facebook, Twitter, Instagram)
- [ ] Saw the survey link at my GP
- [ ] Advert in a shop
- [ ] Advert in community centre
- [ ] Other

If you selected Other, please specify:

Would you be interested in taking part in future research studies with the University of Liverpool, NIHR Health Protection Research Unit in Gastrointestinal Infections?

( ) Yes ( ) No

Please proivide your email:

Please enter a valid email address.

Please provide a valid telephone number:

Please enter a valid phone number.

Page 8: Final page

Thank you for completing this survey.

For more information on the research team please see http://www.hprugi.nihr.ac.uk/

For advice if you have symptoms of diarrhoea and vomiting please visit
https://www.nhs.uk/conditions/diarrhoea-and-vomiting/

ENTER THE PRIZE DRAW FOR A £100 AMAZON VOUCHER

Key for selection options

3 - What is your age in years?
- 16-17
- 18-24
- 25-34
- 35-44
- 45-54
- 55-64
- 65+

4 - What is your highest educational attainment?
- No qualifications
- NVQ/GCSE/O Level
- A levels/BTEC/level 3 diploma
- Degree/PGDips
- Masters
- PhD
- Other

8 - What is your ethnicity?
- White: British
- White: Irish
- White: Gypsy or Irish Traveler
- White: Other
- Mixed/multiple ethnic groups: White and Asian
- Mixed/multiple ethnic groups: White and Black African
- Mixed/multiple ethnic groups: White and Black Caribbean
- Mixed/multiple ethnic groups: Other
- Asian British/Asian: Chinese
- Asian British/Asian: Pakistani
- Asian British/Asian: Indian
- Asian British/Asian: Bangladeshi
- Asian British/Asian: Other

Black British/Black/African/Caribbean: African
Black British/Black/African/Caribbean: Caribbean
Black British/Black/African/Caribbean: Other
Other ethnic group: Arab
Other ethnic group: Other
